# Changes in Modifiable Health Behaviors During the Pandemic and Effects on Mental Health: Evidence From England

**DOI:** 10.1093/geroni/igab046.904

**Published:** 2021-12-17

**Authors:** Giorgio Di Gessa, Paola Zaninotto

**Affiliations:** University College London, London, England, United Kingdom

## Abstract

COVID-19 mitigation efforts (including lockdowns and advice to stay at home as much as possible) are likely to have resulted in changes in health behaviours such as the amount of sleep, physical exercise, alcohol use, and eating. To date, little is known about how and to what extent these changes in health behaviours since the beginning of the pandemic are related to mental health. Using pre-pandemic data from Wave 9 (2018/19) and from two Covid-19 sub-studies (with data collection in June/July and November/December 2020) of the English Longitudinal Study of Ageing, we investigate how changes in health behaviour during the initial months of the pandemic are associated with subsequent mental health among older people. In our regression analyses, we considered depression and anxiety and controlled for pre-pandemic measures of mental health. Between March and June/July 2020, about a third of older people reported less physical activity; one in five less sleep; and one in ten eating less food and drinking more. Compared to respondents who did not change their behaviours, those who reported sleeping and eating both more and less, and who mentioned less physical activity were more likely to report depression and anxiety, even taking into account pre-pandemic mental health. An increase in drinking was also marginally associated with higher depression. Policymakers should encourage older people who have engaged in unhealthier behaviours to modify them to reduce the negative long-term effects on their mental health.

